# The complete chloroplast genome of *Crateva unilocularis* (Capparaceae)

**DOI:** 10.1080/23802359.2021.1878949

**Published:** 2021-03-01

**Authors:** Xin-hua Zheng, Hua-chao Duan, Shi-min Li, Qiong Dong

**Affiliations:** a Key Laboratory of National Forestry and Grassland Administration on Biodiversity Conservation in Southwest China, Kunming, China; b Forestry College, Southwest Forestry University, Kunming, China

**Keywords:** *Crateva unilocularis*, chloroplast genome, phylogenetic analysis

## Abstract

*Crateva unilocularis* is naturally distributed in Southern China, which is an elite natural tree with high edible and medicinal value. In this study, whole chloroplast (cp) genome of *Crateva unilocularis* was assembled and characterized on the basis of Illumina pair-end sequencing data. The complete cp genome was 156,417 bp in length, containing a large single-copy region (LSC) of 85,607 bp and a small single-copy region (SSC) of 18,164 bp, which were separated by a pair of 26,323 bp inverted repeat regions (IRs). The genome contained 128 genes, including 85 protein-coding genes, 35 tRNA genes, and 8 rRNA genes. The overall GC content is 36.32%, while the corresponding values of the LSC, SSC, and IR regions were 33.98, 29.45, and 42.48%, respectively. The maximum-likelihood phylogenetic analysis showed a strong sister relationship with *Crateva tapia*. These findings provide a foundation for further investigation of cp genome evolution in *Crateva unilocularis* and other higher plants.

*Crateva unilocularis* is a deciduous tree species belong to the family Capparaceae which mainly distributed in southwest China (Sha 2006), it is a unique edible and valuable medicinal plant for Chinese medicine (Sha et al. 2008; Dong et al. 2011). It is especially valued for nutrition and medicine because the young leaves are rich in vitamin C, amino acids and proteins (Cheng et al. 2000). It is well known as an edible vegetable by the local ethnic minorities (Li et al. 2018). Apart from nutritional values, it is a timber species and has ecological protection threats (Nai 2005). Digging its genetic information can help to study the species diversity and evolutionary relationships.

Chloroplast genome are widely being used in the DNA barcoding (Dong et al. 2014), phylogenetic relationships, biology and species conservation (Xue et al. 2012). To date, chloroplast genomes have been reported in many species, such as *Origanum vulgare*, *Capsella rubella* and *Schrenkiella parvula* ((Lukas and Novak 2013； Wu 2015； He et al. 2016) and many others. In this paper, we report the complete chloroplast genome sequence of *Crateva unilocularis* based on the Illumina pair-end sequencing data. This will provide benefits for further studies on biological research in the Capparaceae order.

The fresh leaf samples of *Crateva unilocularis* were obtained from Kunming, Yunnan, China (geospatial coordinates: E102°45′55″, N25°03′56″, altitude: 1954 m), a voucher specimen (KUN 1265460) is deposited at the Kunming institute of botany, Chinese Academy of Sciences. Extraction of total genomic DNA was done using magnetic beads plant genomic DNA preparation kit. The DNA samples were stored at the Key Laboratory of National Forestry and Grassland Administration on Biodiversity Conservation in Southwest China, Southwest Forestry University, Kunming, China.

After genomic DNA extraction, a library with the insertion size of 150 bp was constructed and high-through put DNA sequencing was performed on an Illumina Hiseq X plat form. After obtaining approximately 2.93 Gb high-quality clean reads, the raw data were used to assemble a complete Cp genome made by software from GetOrganelle with reference to *Champereia manillana* (Jin et al. 2018). The Geneious R8 (Biomatters Ltd, Auckland, New Zealand) was used to assemble and execute complete cp enzyme labeling annotations. Finally, the Cp DNA sequence of *Crateva unilocularis* was submitted to GenBank (accession number: MT679554).

The complete length of *Crateva unilocularis* cp genome was 156,417 bp (Figure 1), comprising of a large single-copy region (LSC with 85,607 bp) and a small single-copy region (SSC with 18,164 bp), which were separated by a pair of inverted repeats (IRs with 26,323 bp). The overall GC content of genome was 36.32%, the GC content of the LSC (33.98%), and SSC (29.45%) regions were relatively lower than that of the IR regions (42.48%). A total of 128 functional genes were contained in the cp genome, including 85 protein-coding genes (PCG), 8 rRNA genes, and 35 tRNA genes.

**Figure 1.**
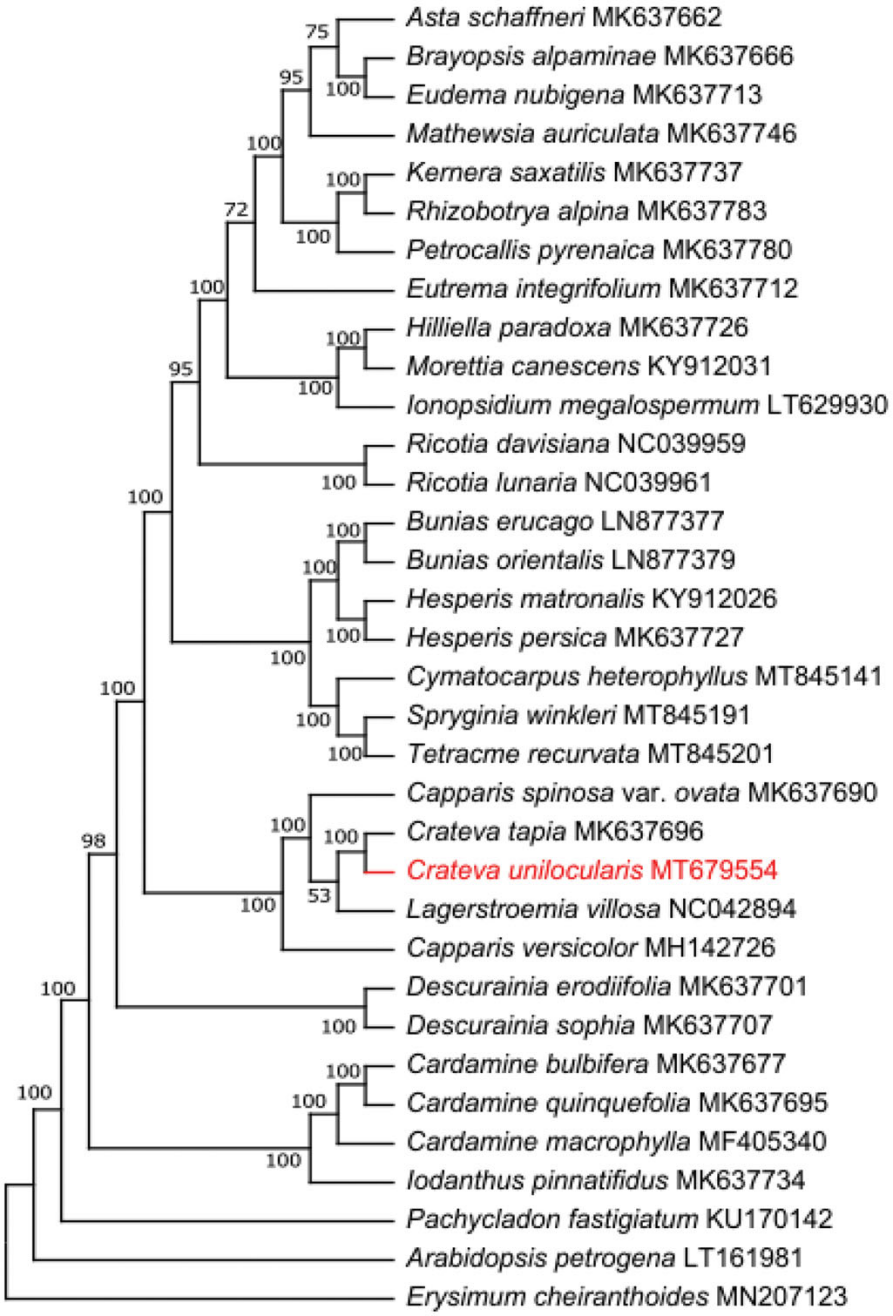
The best maximum likelihood (ML) phylogram inferred from 34 chloroplast genomes in Capparaceae.

To further investigate the phylogenetic position of *Crateva unilocularis*, The maximum-Likelihood (ML) tree was constructed based on complete cp genome sequences of 24 other Capparaceae species using MEGA-X with 1000 bootstrap replicates (Kumar et al. 2016). The result of the phylogenetic analysis showed that *Crateva unilocularis* is closely related to the species of *Crateva tapia*. In conclusion, complete chloroplast genome of *Crateva unilocularis* is decoded for the first time, which will also provide valuable information for the study of the genetic diversity and further biological analysis of *Crateva unilocularis* and Capparaceae.

## Data Availability

The data that support the findings of this study are openly available in [National Center for Biotechnology Information], [https://www.ncbi.nlm.nih.gov/Traces/study/?acc=PRJNA671642], accession number [MT679554].
